# 623. Advancing Fungal Diagnostics with a Transcriptional Approach

**DOI:** 10.1093/ofid/ofad500.689

**Published:** 2023-11-27

**Authors:** Eleanor Young, Roby P Bhattacharyya, Poppy Sephton-Clark, Christina Cuomo

**Affiliations:** The Broad Institute, Cambridge, Texas; Massachusetts General Hospital, Cambridge, Massachusetts; Broad Institute, Cambridge, Massachusetts; Genomic Center for Infectious Diseases at the Broad Institute of MIT and Harvard, Cambridge, Cambridge, Massachusetts

## Abstract

**Background:**

Invasive fungal infections are increasingly common and carry high rates of morbidity and mortality, and rising rates of drug resistance, notably in *Candida auris*. However, there is a notable gap between the development of bacterial identification (ID) and antimicrobial susceptibility testing (AST) diagnostics and a relative lack thereof for fungi.

**Methods:**

For ID, we applied phylogeny-informed rRNA-based strain identification (Phirst-ID) to *Candida* species, using variable regions of the 18S and 28S rRNA of 11 pathogenic *Candida* species. We tested 66 laboratory isolates and 62 clinical blood cultures on the NanoString (Seattle,WA) RNA detection platform. For AST, we leveraged transcriptional differences between susceptible and resistant isolates upon antifungal exposure as a phenotypic measure of susceptibility, agnostic to resistance mechanism. We performed RNA-Seq on susceptible and resistant *C. albicans* treated with fluconazole and *C. auris* treated with the major antifungal classes (voriconazole, micafungin, or amphotericin) at clinical breakpoint concentrations and screened for key transcripts whose antibiotic response best distinguished susceptible from resistant isolates.

**Results:**

From lab culture, *Candida* Phirst-ID distinguished 11 common pathogenic *Candida* species, including *C. auris*, with 100% accuracy. From clinical blood cultures, we correctly identified all 57 monomicrobial *Candida* species, without misidentifying 3 non-*Candida* isolates. For AST, in *C. albicans*,the response of ERG genes to fluconazole exposure cleanly distinguished 8 susceptible from 8 resistant isolates. RNA-seq data from *C. auris* exposed to amphotericin B, micafungin, and voriconazole suggest differential regulation in several key transcripts between susceptible and resistant isolates, and we are investigating their diagnostic utility in NanoString assays.

Transcriptional profiling provides accurate AST in yeast.

**Figure:**
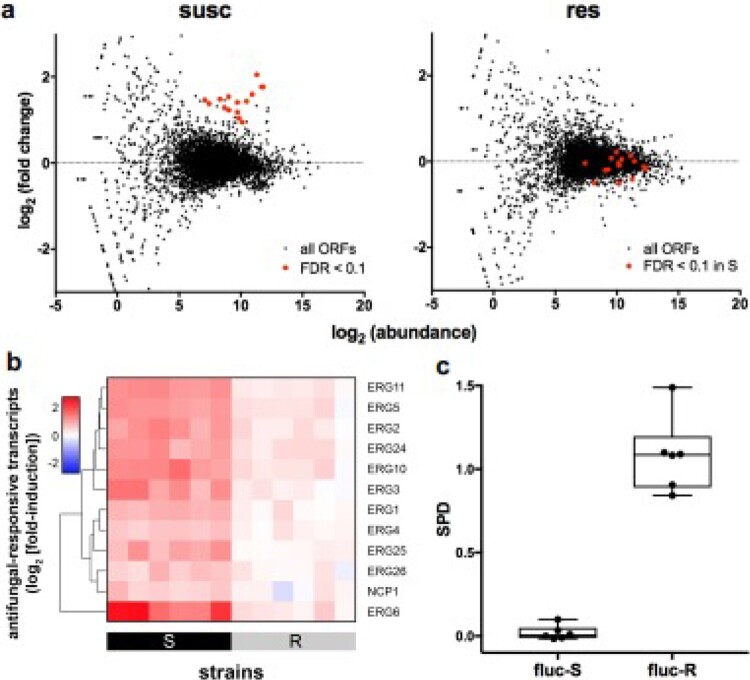

(a) RNA-Seq data showing showing transcriptional response in susceptible (left) but not resistant (right) isolates of C. albicans isolates upon fluconazole exposure. Transcripts with false discovery rate (FDR) < 0.1 in susceptible isolates are shown in red in both plots; no transcripts reach statistical significance in resistant isolates. (b) Heatmaps of normalized NanoString data for 10 fluconazole-responsive transcripts from 12 isolates of C. albicans, arranged by increasing MIC, with AST classification below. (c) One-dimensional projections of NanoString data clearly distinguish C. albicans isolates by fluconazole susceptibility.

**Conclusion:**

Our *Candida* species ID assay is a simple, robust assay that can provide accurate results within hours directly from blood culture bottles which we are extending into a pan-fungal assay tested on clinical specimens. The transcriptional signature of select genes in fluconazole exposed *C. albicans* distinguishes isolates’ susceptibility and we are working to identify transcripts with such behavior in *C. auris*.

**Disclosures:**

**All Authors**: No reported disclosures

